# Conducting the non-inferiority test for the means with unknown coefficient of variation in a three-arm trial

**DOI:** 10.1186/s12874-023-01990-w

**Published:** 2023-08-11

**Authors:** Meng-Chih Lee, Wei-Ya Wu, Hung-Yi Lu, Hsin-Neng Hsieh, Wei-Hwa Wu

**Affiliations:** 1 Taichung Hospital, Ministry of Health and Welfare, Taichung City, Taiwan; 2 College of Management, Chaoyang University of Technology, Taichung City, Taiwan; 3 East District Public Health Center, Taichung City, Taiwan; 4 Department of Statistics and Information Science, Fu Jen Catholic University, New Taipei City, Taiwan; 5 Department of Finance, Ming Chuan University, Taipei City, Taiwan

**Keywords:** Heteroskedasticity, Coefficient of variation, Generalized *p*-value, Non-inferiority test, Searls’ estimator

## Abstract

**Background:**

The non-inferiority test is a reasonable approach to assessing a new treatment in a three-arm trial. The three-arm trial consists of a placebo, reference, and an experimental treatment. The non-inferiority is often measured by the mean differences between the experimental and the placebo groups relative to the mean differences between the reference and the placebo groups.

**Methods:**

To cope with possible estimation distortion due to the allowance of heteroskedasticity, we adjust the measurement of non-inferiority by the incorporation of coefficient of variation (CV) of the experimental, the reference and the placebo groups. In this research, we propose a generalized $p$-value based method (GPV-based method) to facilitate non-inferiority tests for the means with unknown coefficient of variation in a three-arm trial.

**Results:**

The simulation results show that the GPV-based method can not only adequately control type I error rate at nominal level better but also provide power higher than those from Delta method and the empirical bootstrap method, which verifies the feasibility of our adjustment.

**Conclusions:**

We revise the measurement of non-inferiority by deducting the CV of each kind of treatment from the average effect of trials. CVs are included in the non-inferiority explicitly to help prevent possible estimating distortion if heteroskedasticity is allowed. Through the simulation study, the performance of GPV-based method for facilitating non-inferiority tests for the means with unknown CV in a three-arm trial is better than those from empirical bootstrap method and Delta method for small, medium and large sample sizes. Hence, the GPV-based method is recommended to be used to conduct the non-inferiority test for the means with unknown CV in a three-arm trial. The GPV-based method still performs well in non-normality cases.

**Supplementary Information:**

The online version contains supplementary material available at 10.1186/s12874-023-01990-w.

## Background

The goal of a non-inferiority test is to determine whether the experimental treatment is statistically not inferior to the active control in a clinical trial. The three-arm clinical trial for non-inferiority test is validated by the recommendation from U.S. Food and Drug Administration (FDA). The three-arm trial, consisting of a placebo, reference, and an experimental treatment, shows the substantial superiority of the comparator over the placebo which is assessed prior to the comparison of reference and new experiment treatment [1]. Pigeot et al. [2] formulated the problem of non-inferiority test in three-arm trial as a ratio, which is the mean in experimental groups to the mean in reference groups, while deducting the mean in placebo groups respectively. Under a given threshold *α*_0_ (say 0.8), if the alternative hypothesis holds true, then it implies that the efficacy of the experimental group relative to that of the placebo group is more than *α*_0_×100% of the efficacy of the reference compound relative to that of the placebo group. Under normality and homogeneous variance assumption, Pigeot et al. [2] developed a test statistic in t-distribution to construct the confidence interval for the hypothesis of ratio by Fieller’s method. Meanwhile, Hasler et al. [3] derived a *t*-distributed test statistic under the variance heteroscedasticity assumption and the confidence intervals based on Fieller’s method.

In the above literatures, the test statistic of a non-inferiority test in the three-arm trial is the sample mean difference between the experimental and placebo groups denominated by that between the reference and placebo groups in the three-arm trial. It’s well perceived that the sample mean is an unbiased estimator for population mean. Casting aside the unbiasedness, Searls [4] proposed an estimator for mean that includes a known coefficient of variation (CV) in advance, which has a minimum mean square error. In Wu and Hsieh [5], through estimating the population mean of treatment effects in a three-arm rial by Searls’ estimator rather than traditional simple sample mean, they show that Searls’ estimator performs better, in terms of empirical size and empirical power. Thangjai et al. [6] derives the expectation and variance of Searls’ estimator (with unknown CV). Moreover, Thangjai et al. [6] also constructed the confidence intervals for mean and difference of means of normal distributions with unknown coefficients of variation. In this study, we try to use the concept of Thangjai et al. [6] to propose the non-inferiority test procedure in the three-arm trial in which the non-inferiority is measured as the mean difference with unknown coefficient of variation between the experimental and the placebo groups relative to that between the reference and the placebo groups. Since the assumption of heterogeneous variances complicates the distributions of estimators of the difference between the mean with unknown CV of the experimental and the placebo groups relative to that between the reference and the placebo groups, it is a challenge to measure the non-inferiorities of new treatments in the three-arm clinical trial. Consequently, we propose the generalized $p$-value based method (hereafter GPV-based method) that is the statistical test procedure to assess the non-inferiority test in the three-arm trial under heterogeneous variances assumption with unknown coefficient of variation of treatments.

Typically, in the three-arm non-inferiority tests, variances of the effects of trials are assumed to be homogeneous. But if the variances are heterogeneous, the impacts of heteroskedasticity on the test results are evaluated less times. The heteroskedasticity is an issue frequently encountered in the field of econometrics, which results in the problem of biased variance estimates and hence distorts the results of hypothesis tests such as CHOW’s coefficient stability test, Student’s *t*-test, and Fisher’s *F*-test [7]. Though earlier researches use the tests on variances to detect whether heteroskedasticity exists in the model, Li and Yao [8] and Tovohery et al. [7] use the coefficient of variation (CV) to detect such problem. Inspired by Searls [4], in this research, we explicitly incorporate CV into the mean of the observations of trials, that is, substituting the population mean by Searls’ estimator in measuring the non-inferiority, to mitigate the impacts of heteroskedasticity on the test results.

Tsui and Weerahandi [9] explicitly defined the generalized test variables (GTVs), showing that the generalized $p$-value (GPV) is an exact probability in an extreme region accordingly. Based on their contribution, Tsui and Weerahandi [9] demonstrated that how small sample solution can be provided with GPVs to the cases where nuisance parameters emerge such that testing procedures are difficult to be conducted. Since the proposal of the idea of GPVs, they are applied to several hypothesis test subjects. For instance, Liao et al. [10, 11] applied the GPV to tolerance intervals; McNally et al. [12] conducted individual and population bioequivalence tests by GPVs; Mathew and Webb [13] constructed the GPVs and GCIs for variance components; Gamage [14] applied GPVs to MANOVA; with the concept of GPVs, Li et al. [15] measured the difference in paired partial area under the receiver operating characteristic (ROC) curves to construct a non-inferiority test for diagnostic accuracy. Gamalo et al. [16] proposed a GPV approach to assessing the non-inferiority in a three-arm trial, in which the hypothesis test taken into account is the same as those in Hasler et al. [3].

The article is organized as follows. The statistical problem of the non-inferiority hypothesis test with unknown CV in three-arm trial is formulated and the test procedures implemented in bootstrap method and Delta method are derived in the second part of the article. In addition, we propose the GPV-based test for the ratio of mean differences which explicitly incorporating the unknown CV to assess the non-inferiority in a three-arm trial in the second part of the article. Furthermore, the empirical size and power of the proposed testing procedures are examined in simulation studies under a variety of scenarios. The proposed method is applied to a numerical example in the literature. Conclusion and some remarks are drawn in finally.

## Methods

Let the clinical observations of experimental treatment, reference, and placebo groups be respectively denoted as $X_{E,i}$,$X_{R,j}$ and $X_{P,k}$, which are mutually independent and normally distributed with expectations $\mu_{E}$, $\mu_{R}$ and $\mu_{P}$, and unknown variances $\sigma_{E}^{2}$,$\sigma_{R}^{2}$ and $\sigma_{P}^{2}$, respectively. Since the variance in the reference group is the gold standard in the three-arm trial, to allow for a fair standard of non-inferiority test, in this study, we assume that the variance of the experimental treatment group is equal to that of the reference group, but which is heterogeneous to that of the placebo group. Specifically, $X_{{E,{\kern 1pt} i}} \sim N\left( {\mu_{E} ,\sigma_{E}^{2} } \right),{\kern 1pt} {\kern 1pt} i = 1, \ldots ,n_{E}$; $X_{{R,{\kern 1pt} j}} \sim N\left( {\mu_{R} ,\sigma_{R}^{2} } \right),{\kern 1pt} j = 1, \ldots ,n_{R}$; and $X_{{P,{\kern 1pt} k}} \sim N\left( {\mu_{P} ,\sigma_{P}^{2} } \right),{\kern 1pt} {\kern 1pt} k = 1, \ldots ,n_{P}$, where $\sigma_{E}^{2} = \sigma_{R}^{2}$, and $n_{E}$,${\kern 1pt} {\kern 1pt} n_{R}$ and $n_{P}$ can be unequal. Firstly, establishing the statistical testing problem

$$H_{0} :\theta_{E} - \theta_{R} \le \delta_{0}\ \mathrm{versus}\ H_{1} :\theta_{E} - \theta_{R} > \delta_{0}$$

where $\theta_{E} = \frac{{n_{E} \mu_{E} }}{{n_{E} + \left( {{{\sigma_{E}^{2} } \mathord{\left/ {\vphantom {{\sigma_{E}^{2} } {\mu_{E}^{2} }}} \right. \kern-0pt} {\mu_{E}^{2} }}} \right)}}$, $\theta_{R} = \frac{{n_{R} \mu_{R} }}{{n_{R} + \left( {{{\sigma_{R}^{2} } \mathord{\left/ {\vphantom {{\sigma_{R}^{2} } {\mu_{R}^{2} }}} \right. \kern-0pt} {\mu_{R}^{2} }}} \right)}}$, $\theta_{P} = \frac{{n_{P} \mu_{P} }}{{n_{P} + \left( {{{\sigma_{P}^{2} } \mathord{\left/ {\vphantom {{\sigma_{P}^{2} } {\mu_{P}^{2} }}} \right. \kern-0pt} {\mu_{P}^{2} }}} \right)}}$, where $\sigma_{E}^{2} = \sigma_{R}^{2}$ and $\delta_{0}$ is a relevant non-inferiority threshold. For $\xi_{0} \in (0,1)$, we specify $\delta_{0}$ as a proportion of the difference between $\theta_{E}$ and $\theta_{R}$ by $\delta_{0} = (\xi_{0} - 1)(\theta_{R} - \theta_{P} )$. Then rewriting the hypothesis based on the ratio of the differences in means with unknown CV yields

1 $$H_{0} :\frac{{\theta_{E} - \theta_{P} }}{{\theta_{R} - \theta_{P} }} \le \xi_{0} {\kern 1pt} {\text{versus}}{\kern 1pt} {\kern 1pt} H_{1} :\frac{{\theta_{E} - \theta_{P} }}{{\theta_{R} - \theta_{P} }} > \xi_{0}$$

where $\xi_{0}$ represents the effectiveness threshold between 0 and 1. The value of $\theta_{R} - \theta_{P}$ is necessarily greater than 0. Because the threshold value $\xi_{0}$ is defined as a proportion of the difference $\theta_{R} - \theta_{P}$, it is important to select proper reference or positive control. In this way, the evaluation of the non-inferiority in the three-arm trial is specified as a ration of difference in population mean with unknown CV, as is discusses in the background of the text.

### Empirical bootstrap method

The bootstrap method has become a widely used technique for statistical inference problem in which either the underlying distributional assumptions are not normal distribution, or the sample statistic is not feasible to derive its distribution under the null hypothesis (Efron and Tibshirani [17]). Now that the variance of experimental treatment group is equal to that of reference group (which is heterogeneous to that of the placebo group), we use the residual method to construct the empirical bootstrap procedure to assess the non-inferiority of a new treatment in a three-arm trial. The residual method is somewhat similar to the percentile method, except that it is based on the bootstrap distribution of residuals from the original estimate [18]. The empirical bootstrap procedure can be obtained as follows.

- Step1**:** Suppose that ${\mathbf{x}}_{E} = \left( {x_{E,1} , \ldots ,x_{{E,n_{E} }} } \right)$,${\mathbf{x}}_{R} = \left( {x_{R,1} , \ldots ,x_{{R,n_{R} }} } \right)$ and ${\mathbf{x}}_{P} = \left( {x_{P,1} , \ldots ,x_{{P,n_{P} }} } \right)$ denote the clinical observations for experimental, reference and placebo groups, respectively. Generate a bootstrap sample ${\mathbf{x}}^{*b} = \left( {{\mathbf{x}}_{E}^{*b} ,{\mathbf{x}}_{R}^{*b} ,{\mathbf{x}}_{P}^{*b} } \right)$ with replacement from the original sample ${\mathbf{x}} = \left( {{\mathbf{x}}_{E} ,{\mathbf{x}}_{R} ,{\mathbf{x}}_{P} } \right)$ and draw samples with replacement from each group with sample sizes $n_{E}$, $n_{R}$ and $n_{P}$, respectively.
- Step 2**: **Compute $\hat{\xi }^{*b} = \frac{{\widehat{\theta }_{E}^{*b} - \widehat{\theta }_{P}^{*b} }}{{\widehat{\theta }_{R}^{*b} - \widehat{\theta }_{P}^{*b} }}$ from data ${\mathbf{x}}^{*b}$ and $e^{*b} = \hat{\xi }^{*b} - \widehat{\xi }$ is calculated for each bootstrap sample, where $\hat{\xi }$ is the estimate from the original data.
- Step 3**:** Repeat step1 and step2 process $b = 1, \cdots ,B$ times independently.
- Step 4**:** Let $e_{(1 - \alpha )100\% }^{*b}$ be the $(1 - \alpha )100\%$ quantile of the bootstrap values of $e^{*b}$, and compute the $L_{{\widehat{\xi }^{b} }} = \widehat{\xi } - e_{(1 - \alpha )100\% }^{*b}$.

Then, non-inferiority can be claimed if $L_{{\widehat{\xi }^{b} }} > \xi_{0}$.

### Delta method

Let $\xi_{1} = \theta_{E} - \theta_{P}$ be the difference of population mean with unknown CV in experimental group and placebo group and let $\xi_{2} = \theta_{R} - \theta_{P}$ be the difference of population mean with unknown CV in reference group and placebo group. Therefore, the expectations and variances of $\hat{\xi }_{1}$ and $\hat{\xi }_{2}$ can be obtained by Thangjai [6]. The Delta method is proposed in Dorfman [19]. Such method is the result of the application of the concept of Taylor's theorem (series expansion) to construct the normal distribution of the estimators in complex forms asymptotically. Accordingly, the threshold, $\widehat{\xi } = \frac{{\widehat{\xi }_{1} }}{{\widehat{\xi }_{2} }}$ is distributed asymptotically as.

$$\widehat{\xi }\mathop \sim \limits_{asymp} N\left( {E(\widehat{\xi })\,,\,Var(\widehat{\xi })} \right),$$

where

$$E(\widehat{\xi }) = E\left( {\frac{{\widehat{\xi }_{1} }}{{\widehat{\xi }_{2} }}} \right) \approx \frac{{\mu_{{\xi_{1} }} }}{{\mu_{{\xi_{2} }} }},$$

$$Var(\widehat{\xi }) = Var\left( {\frac{{\widehat{\xi }_{1} }}{{\widehat{\xi }_{2} }}} \right) \approx \left( {\frac{{\mu_{{\xi_{1} }} }}{{\mu_{{\xi_{2} }} }}} \right)^{2} \left( {\frac{{Var(\widehat{\xi }_{1} )}}{{\mu_{{\xi_{1} }}^{2} }} + \frac{{Var(\widehat{\xi }_{2} )}}{{\mu_{{\xi_{2} }}^{2} }} - 2\frac{{Cov(\widehat{\xi }_{1} ,\widehat{\xi }_{2} )}}{{\mu_{{\xi_{1} }} \mu_{{\xi_{2} }} }}} \right).$$

When the null hypothesis holds, for the non-inferiority hypothesis test in terms of population mean with unknown CV as shown in (1), the rejection region constructed under Delta method is.

$$C_{Delta\;method} = \left\{ {\widehat{\xi } - z_{\alpha } \sqrt {Var(\widehat{\xi })} > \xi_{0} } \right\},$$

where $z_{\alpha }$ denotes the upper $\alpha$ critical point of the standard normal distribution.

### The GPV-based method

Suppose ${\mathbf{X}}$ to be the random variable whose PDF is $f({\mathbf{X}};\zeta )$, where $\zeta = (\xi ,\eta )$. The $\xi$ is parameter of interest such that $\xi = \frac{{\theta_{E} - \theta_{P} }}{{\theta_{R} - \theta_{P} }}$ and $\eta$ denotes a vector of nuisance parameters. Let ${\mathbf{x}}$ be the observed value of the random variable ${\mathbf{X}}$. The statistic $T = T\left( {{\mathbf{X}};{\mathbf{x}},\zeta } \right)$ is said to be a generalized test variable if the following three properties hold.

- Property A: Fixing ${\mathbf{x}}$ and let $\zeta = (\xi_{0} ,\eta )$, the distribution of $T({\mathbf{X}};{\mathbf{x}},\zeta )$ is independent of nuisance parameters $\eta$.
- Property B: The observation of $T({\mathbf{X}};{\mathbf{x}},\zeta )$, $t_{obs} = T\left( {{\mathbf{x}};{\mathbf{x}},\zeta } \right)$, does not dependent on unknown parameters.
- Property C: For given ${\mathbf{x}}$ and $\eta$, $P\left( {T({\mathbf{X}};{\mathbf{x}},\zeta ) \ge t} \right)$ is either stochastically increasing or decreasing in $\xi$ for any given $t$.

Without loss of generality, considering the following hypothesis: to test $H_{0} :\xi \le \xi_{0}$ versus $H_{1} :\xi > \xi_{0}$, where $\xi_{0}$ is a specified value. If $T$ is stochastically increasing in $\xi$, then the generalized $p$-value can be defined as.

$$p = \mathop {\sup }\limits_{{\xi \le \xi_{0} }} P\left( {T({\mathbf{X}};{\mathbf{x}},\xi ,\eta ) \ge t_{obs} } \right) = P\left( {T({\mathbf{X}};{\mathbf{x}},\xi_{0} ,\eta ) \ge t_{obs} } \right) = P\left( {T \ge t_{obs} |\xi_{0} } \right),$$

where $$t_{obs} = T({\mathbf{x}};{\mathbf{x}},\xi_{0} ,\eta )$$.

For the test with a significance level $\alpha$, if $p < \alpha$, then we have confidence to reject $H_{0}$. The generalized test variable $T$ is often computed by using Monte-Carlo algorithm, due to the complexity of the exact distribution.

In the following, we use the concept of generalized pivotal quantity (GPQ) by Weerahandi [20] to develop the required generalized test variables (GTVs) to assessment non-inferiority of a new treatment in a three-arm trial measured as a ratio of difference in mean with CV of each treatment. For developing the GTV for hypothesis test in (1), we first define GPQs for $\mu_{E}$, $\mu_{R}$, $\mu_{P}$,$\sigma_{E}^{2}$, $\sigma_{R}^{2}$, $\sigma_{P}^{2}$, $\theta_{E}$, $\theta_{R}$ and $\theta_{P}$ as

2 $$R_{{\mu_{E} }} = \overline{x}_{E} - Z_{E} \sqrt {\frac{{(n_{E} - 1)s_{pooled}^{2} }}{{n_{E} U_{E} }}}$$

3 $$R_{{\mu_{R} }} = \overline{x}_{R} - Z_{R} \sqrt {\frac{{(n_{R} - 1)s_{pooled}^{2} }}{{n_{R} U_{R} }}}$$

4 $$R_{{\mu_{P} }} = \overline{x}_{P} - Z_{P} \sqrt {\frac{{(n_{P} - 1)s_{P}^{2} }}{{n_{P} U_{P} }}}$$

5 $$R_{{\sigma_{E}^{2} }} = \frac{{(n_{E} - 1)s_{pooled}^{2} }}{{U_{E} }}$$

6 $$R_{{\sigma_{R}^{2} }} = \frac{{(n_{R} - 1)s_{pooled}^{2} }}{{U_{R} }}$$

7 $$R_{{\sigma_{P}^{2} }} = \frac{{(n_{P} - 1)s_{P}^{2} }}{{U_{P} }}$$

8 $$R_{{\theta_{E} }} = \frac{{n_{E} R_{{\mu_{E} }} }}{{n_{E} + R_{{{{\sigma_{E}^{2} } \mathord{\left/ {\vphantom {{\sigma_{E}^{2} } {\mu_{E}^{2} }}} \right. \kern-0pt} {\mu_{E}^{2} }}}} }}$$

9 $$R_{{\theta_{R} }} = \frac{{n_{R} R_{{\mu_{R} }} }}{{n_{R} + R_{{{{\sigma_{R}^{2} } \mathord{\left/ {\vphantom {{\sigma_{R}^{2} } {\mu_{R}^{2} }}} \right. \kern-0pt} {\mu_{R}^{2} }}}} }}$$

10 $$R_{{\theta_{P} }} = \frac{{n_{P} R_{{\mu_{P} }} }}{{n_{P} + R_{{{{\sigma_{P}^{2} } \mathord{\left/ {\vphantom {{\sigma_{P}^{2} } {\mu_{P}^{2} }}} \right. \kern-0pt} {\mu_{P}^{2} }}}} }}$$

Note that $Z_{E} \sim N(0,1)$, $Z_{R} \sim N(0,1)$, $Z_{P} \sim N(0,1)$, $U_{E} \sim \chi^{2} (n_{E} - 1)$, $U_{R} \sim \chi^{2} (n_{R} - 1)$, $U_{P} \sim \chi^{2} (n_{P} - 1)$, $\overline{x}_{E}$, $\overline{x}_{R}$ and $\overline{x}_{P}$ be the observed values of $\overline{X}_{E}$, $\overline{X}_{R}$ and $\overline{X}_{P}$, $s_{E}^{2}$, $s_{R}^{2}$ and $s_{P}^{2}$ be the observed values of $S_{E}^{2}$, $S_{R}^{2}$ and $S_{P}^{2}$. In addition, we use pooled estimator $S_{pooled}^{2}$ to estimate both $\sigma_{E}^{2}$ and $\sigma_{R}^{2}$. The pooled estimator is defined as $S_{pooled}^{2} = {{\left( {(n_{E} - 1)S_{E}^{2} + (n_{R} - 1)S_{R}^{2} } \right)} \mathord{\left/ {\vphantom {{\left( {(n_{E} - 1)S_{E}^{2} + (n_{R} - 1)S_{R}^{2} } \right)} {\left( {n_{E} + n_{R} - 2} \right)}}} \right. \kern-0pt} {\left( {n_{E} + n_{R} - 2} \right)}}$, and the $s_{pooled}^{2}$ be the observed value of $S_{pooled}^{2}$. Moreover, $Z_{E}$, $Z_{R}$, $Z_{P}$, $U_{E}$, $U_{R}$ and $U_{P}$ are mutually independent.

The GPQ of $\xi = \frac{{\theta_{E} - \theta_{P} }}{{\theta_{R} - \theta_{P} }}$ can thus be defined as

11 $$R_{\xi } = R_{{\frac{{\theta_{E} - \theta_{P} }}{{\theta_{R} - \theta_{P} }}}} = \frac{{R_{{\theta_{E} }} - R_{{\theta_{P} }} }}{{R_{{\theta_{R} }} - R_{{\theta_{P} }} }}$$

Hence, we can construct a GTV for $\xi$ given by

12 $$T_{\xi } = T\left( {{\mathbf{X}}_{E} ,{\mathbf{X}}_{R} ,{\mathbf{X}}_{P} ;{\mathbf{x}}_{E} ,{\mathbf{x}}_{R} ,{\mathbf{x}}_{P} ,\xi } \right) = R_{\xi } - \xi$$

Given the observed data, the observed value of $R_{\xi }$ is equal to $\xi$ and $R_{\xi }$ has the distribution that is free of parameters. Hence, the distribution of $T_{\xi }$ does not depend on nuisance parameters for any given value of $\xi = \xi_{0}$, and that the observation of $T_{\xi }$ is equal to zero. Consequently, Property A and Property B are satisfied. Furthermore, the distribution function of $T_{\xi }$ can be expressed as

13 $$P\left( {T_{\xi } \le t} \right) = P\left( {R_{\xi } \le t + \xi } \right)$$

Since the distribution function of $T_{\xi }$ is stochastically increasing in $\xi$, Property C is also satisfied. By definition, $T_{\xi }$ is a GTV of $\xi$. To test the hypothesis $H_{0} :\xi \le \xi_{0} \quad versus\quad H_{1} :\xi > \xi_{0}$, the following Monte-Carlo algorithms are provided to derive the required GPV.

- Step 1: Choose Monte-Carlo samples large enough, e.g., $$H = 10000$$$$10000$$. For each $$h$$, $$1 \le h \le H$$, generate three pairs of random outcomes from mutually independent chi-square distributions, $$U_{E}$$, $$U_{R}$$ and $$U_{P}$$ (with $$n_{E} - 1$$, $$n_{R} - 1$$ and $$n_{P} - 1$$ degrees of freedom) respectively, and standard normal variables $$Z_{E}$$, $$Z_{R}$$ and $$Z_{P}$$.
- Step 2: Use (2)- (10) to calculate $R_{{\mu_{E} }}$, $R_{{\mu_{R} }}$, $R_{{\mu_{P} }}$, $R_{{\sigma_{E}^{2} }}$, $R_{{\sigma_{R}^{2} }}$, $R_{{\sigma_{P}^{2} }}$, $R_{{\theta_{E} }}$, $R_{{\theta_{R} }}$ and $R_{{\theta_{P} }}$.
- Step 3: Calculate $R_{\xi ,h}$ from (11).
- Step 4: Finally, $T_{\xi ,h}$ can be calculated from (12), given $\xi_{0}$.

Since $T_{\xi }$ is stochastically increasing in $\xi$ and the observed value of $T_{\xi }$ is equal to zero, the GPV is thus estimated by $p = {{\sum\nolimits_{h = 1}^{H} {I\left( {T_{\xi ,h} \le 0} \right)} } \mathord{\left/ {\vphantom {{\sum\nolimits_{h = 1}^{H} {I\left( {T_{\xi ,h} \le 0} \right)} } H}} \right. \kern-0pt} H}$. Under significance level $\alpha$, the null hypothesis $H_{0} :\frac{{\theta_{E} - \theta_{P} }}{{\theta_{R} - \theta_{P} }} \le \xi_{0}$ in (1) is rejected whenever $p < \alpha$.

## Results

To evaluate the efficacy of the proposed method, three sets of simulation studies are conducted. First, the empirical sizes from GPV-based method are compared with those from the Delta method and empirical bootstrap method in various finite sample sizes. Second, we evaluate the empirical power among the three tests and compare the performance of the proposed GPV-based method with that of other two tests. Third, we show that GPV-based method can be well applied to non-normality cases.

### Simulation study I: type I error rate

We conducted a simulation study of the type I error rates under GPV-based, Delta and empirical bootstrap methods. The non-inferiority limit is chosen as $\xi_{{0}} { = 0}{\text{.8}}$. We consider the following three cases of $\Delta { = }\mu_{R} - \mu_{P}$: (i) $\Delta = 9$; (ii) $\Delta = 15$ and (iii) $\Delta = 20$. We consider the allocations of 3:2:1 of the total sample size $n$ for experimental, reference and placebo group, so the total sample sizes will choose as follows: $n$ = 60, 90,120,480 and 900, respectively. For cases (i)-(iii), the population mean of placebo group ($\mu_{P}$) is set to be 16.5. The population mean of experimental group is $\mu_{E} = \xi_{0} \times \Delta + \mu_{P}$ under all scenarios. For case (i)-(iii), we consider setting $\tau_{R} = {{\sigma_{R}^{2} } \mathord{\left/ {\vphantom {{\sigma_{R}^{2} } {\sigma_{E}^{2} }}} \right. \kern-0pt} {\sigma_{E}^{2} }}$ to be 1 and $\tau_{P} = {{\sigma_{P}^{2} } \mathord{\left/ {\vphantom {{\sigma_{P}^{2} } {\sigma_{E}^{2} }}} \right. \kern-0pt} {\sigma_{E}^{2} }}$ to be 0.5, 1.0 and 2.0, respectively. In this way, we keep variances of experimental and reference treatments homogeneous, while allowing heteroskedasticity for placebo group. In this simulation study, the standard deviation of placebo group ($\sigma_{P}$) is set to be 7.5, and the standard deviation of reference group ($\sigma_{R}$), as well as the standard deviation of experimental group ($\sigma_{E}$), are both equal to ${{\sigma_{p} } \mathord{\left/ {\vphantom {{\sigma_{p} } {\sqrt {\tau_{p} } }}} \right. \kern-0pt} {\sqrt {\tau_{p} } }}$. In addition, given any pair of $(\mu_{i} ,\sigma_{i} )$, $i = E,R,P$, $\theta_{i}$ and hence $\theta_{E} - \theta_{P}$, $\theta_{R} - \theta_{P}$ can be derived.

Under each parameter specification, the simulation data are independently generated 10,000 times. The empirical size and power are computed by the proportion of the 10,000 simulated $p$-values that are less than 5% (significance level). Given the above nominal significance level and simulation random samples, if a testing procedure can adequately control the size at the 5% nominal level, then the empirical sizes should fall into (0.0457, 0.0543). In this simulation study, for each sample, 5000 GPQs are constructed, and 1000 bootstrap samples are drawn. We display the simulation results in Table 1.

**Table 1:** The type I error rates for testing non-inferiority with non-inferiority limit = 0.8 in $\tau_{R} = 1$, $\mu_{R} - \mu_{P} =$ 9, 15 and 20, respectively

$\mu_{R} - \mu_{P}$	$\tau_{P}$	$n$	GP	DM	EB
9	0.5	60	0.0497	0.0001	0.0001
		90	0.0503	0.0001	0.0003
		120	0.0506	0.0002	0.0009
		480	0.0511	0.0016	0.0082
		900	0.0518	0.0038	0.0264
	1.0	60	0.0486	0.0001	0.0015
		90	0.0493	0.0002	0.0018
		120	0.0498	0.0002	0.0045
		480	0.0501	0.0003	0.0318
		900	0.0506	0.0004	0.0436
	2.0	60	0.0488	0.0001	0.0091
		90	0.0499	0.0001	0.0127
		120	0.0504	0.0002	0.0264
		480	0.0506	0.0002	0.0382
		900	0.0511	0.0003	0.0436
15	0.5	60	0.0475	0.0008	0.0018
		90	0.0481	0.0009	0.0055
		120	0.0482	0.0019	0.0091
		480	0.0499	0.0036	0.0273
		900	0.0502	0.0043	0.0364
	1.0	60	0.0493	0.0004	0.0155
		90	0.0501	0.0004	0.0191
		120	0.0505	0.0007	0.0218
		480	0.0508	0.0007	0.0423
		900	0.0514	0.0015	0.0445
	2.0	60	0.0500	0.0001	0.0327
		90	0.0503	0.0002	0.0364
		120	0.0504	0.0002	0.0373
		480	0.0507	0.0003	0.0455
		900	0.0512	0.0004	0.0473
20	0.5	60	0.0495	0.0021	0.0073
		90	0.0503	0.0025	0.0127
		120	0.0511	0.0032	0.0164
		480	0.0514	0.0047	0.0355
		900	0.0516	0.0058	0.0464
	1.0	60	0.0487	0.0006	0.0264
		90	0.0493	0.0010	0.0291
		120	0.0504	0.0010	0.0303
		480	0.0510	0.0012	0.0455
		900	0.0513	0.0015	0.0467
	2.0	60	0.0486	0.0001	0.0418
		90	0.0490	0.0001	0.0436
		120	0.0502	0.0002	0.0445
		480	0.0509	0.0003	0.0464
		900	0.0515	0.0005	0.0477

$n$ The total sample sizes, *GP* The GPV-based method, *DM* The Delta method and *EB* The empirical bootstrap method

Table 1 presents the results of the type I error rates simulation based on the ratio of population mean differences with unknown coefficients of variation for assessing non-inferiority of a new treatment in a three-arm trial in the presence of heteroscedasticity with non-inferiority limit of 0.8 under normal assumption. The simulation results lead us to the following conclusions.

1. In Table 1, the range of the type I error rates of the GPV-based method is given by (0.0475,0.0518). This range is within (0.0457, 0.0543), and most of the type I error rates of the GPV-based method are quite close to nominal value of 0.05. Therefore, the test procedure of the GPV-based method can maintain type I error rate close to the nominal level of 5% adequately.
2. In addition, from Table 1, the range of the type I error rates from Delta method is (0.0001,0.0058). The ranges of the type I error rates of the Delta method are all outside the range of (0.0457, 0.0543), and all of which are far less than nominal value of 0.05. One may observe that Delta method is quite conservative. However, in some extreme cases (not shown in Table 1), such as $\tau_{p} = 0.01$, and $n = 96,000$, Delta method controls type I error rate much better, and the difference in power between GPV-based and Delta methods shrinks. Apparently, the extreme cases are infeasible for practical clinical application.
3. On the other hand, the range of the type I error rates from the empirical bootstrap method is (0.0001,0.0477). There are only 5 out of 45 (11.1%) empirical sizes from the empirical bootstrap method fall within (0.0457, 0.0543). As a result, the test procedure by the empirical bootstrap method is quite conservative, except when $\mu_{R} - \mu_{P} = 20$, $n \ge 480$, $\tau_{R} = {1}$ and $\tau_{P} = 2$. As the mean difference between reference and placebo groups gets larger, the bootstrap method controls type I error rate better.

Taken as a whole, the GPV-based method performs extremely well in most cases, and it clearly controls the sufficient the type I error rates better, especially in the small sample cases.

### Simulation study II: empirical power

**Fig.1:**
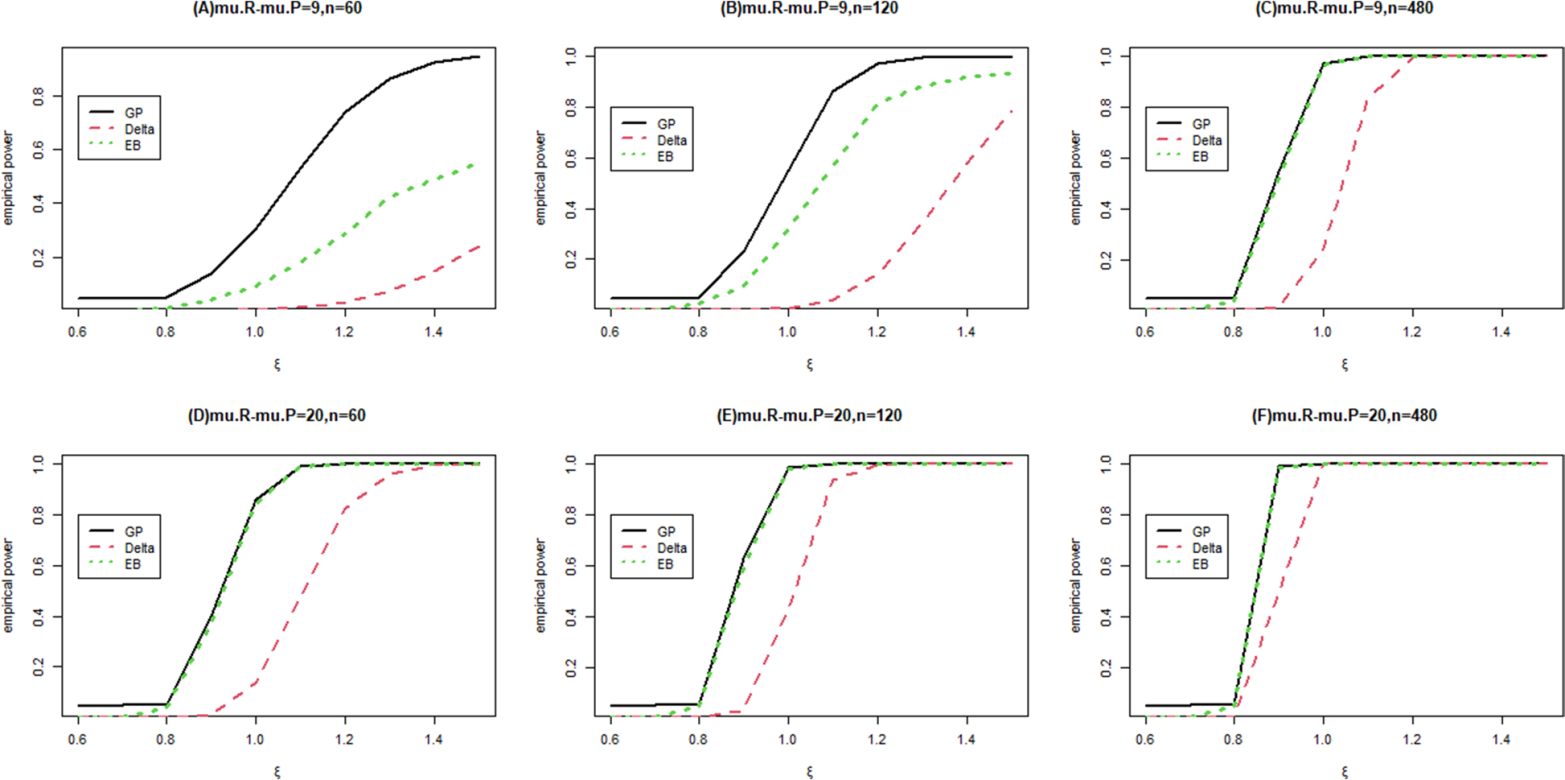
The power functions of GPV-based method (GP), Delta (Delta method) and Empirical bootstrap method (EB). Panel (**A**) represents the power functions when $\mu_{R} - \mu_{P} = 9$ and $n = 60$; Panel (**B**) represents the power functions when $\mu_{R} - \mu_{P} = 9$ and $n = 120$; Panel (**C**) represents the power functions when $\mu_{R} - \mu_{P} = 9$ and $n = 480$;Panel (**D**) represents the power functions when $\mu_{R} - \mu_{P} = 20$ and $n = 60$; Panel (**E**) represents the power functions when $\mu_{R} - \mu_{P} = 20$ and $n = 120$; Panel (**F**) represents the power functions when $\mu_{R} - \mu_{P} = 20$ and $n = 480$. The significance level of the non-inferiority test is set to be 0.05

To study the empirical power of the GPV-based method, we consider a simulation in the case of $\mu_{E} - \mu_{P} = 9$ and $\mu_{E} - \mu_{P} = 20$; $\tau_{R} = 1$ and $\tau_{P} = 2$; sample size = 60,120 and 480. We allocate total sample for experimental, reference and placebo group by $n_{E}$: $n_{R}$: $n_{P}$ = 3: 2: 1. The non-inferiority limit is also chosen as $\xi_{0} = 0.8$, and the significance level is set to be 0.05 as well. For each combination of parameter specification, 10,000 random samples are generated. For each random sample, 5000 GPQs are constructed, and 1000 samples are drawn for bootstrap method. The results of the empirical power curves are provided in Fig. 1.

Figure 1 provides the power of the simulation by GPV-based method, the Delta method, and the empirical bootstrap method. In Fig. 1, when the mean difference of reference and placebo groups is 9, the GPV-based method is uniformly more powerful than the Delta method and the empirical bootstrap method. Figure 1 shows the power curves as a function of $\xi = \frac{{\theta_{E} - \theta_{P} }}{{\theta_{R} - \theta_{P} }}$ for total sample sizes 60,120 and 480, respectively. The power increases with the increasing values of $\xi$ and with the increasing total sample sizes. However, when the mean difference of reference and placebo groups is 20, the empirical power curves of the GPV-based method and the empirical bootstrap method quite overlap when $\xi$ is larger than 0.9. Therefore, when the mean difference of reference and placebo groups is equal to 9, the performance of empirical power by using GPV-based method is better than those of the Delta method and the empirical bootstrap method. On the other hand, the performance of the empirical bootstrap method is as good as that of GPV-based method when the mean difference of reference and placebo groups is equal to 20 and sample size exceeds 60. In sum, the GPV-based method performs relatively better when the mean difference of reference and placebo groups and the sample size are small.

### Simulation study III: non-normality cases

In this section, we consider two non-normal distributions, i.e.,log-normal and gamma distributions to study the robustness of the GPQ-based method. When the probability distribution of the population is assumed to be log-normal distribution, let $$X_{i} \;,\;i = E,\;R,\;P$$ be mutually independent with means $$\ln (\mu_{i} ) - \frac{1}{2}\ln \left( {\frac{{\sigma_{i}^{2} }}{{\mu_{i}^{2} }} + 1} \right)$$ and unknown variances $$\ln \left( {\frac{{\sigma_{i}^{2} }}{{\mu_{i}^{2} }} + 1} \right)$$, respectively. When $$X_{i} \;$$ belongs to the gamma distribution, denote $$X_{i} \;$$ by $$gamma\left( {\gamma_{i1} = \frac{{\mu_{i}^{2} }}{{\sigma_{i}^{2} }}\;,\;\gamma_{i2} = \frac{{\sigma_{i}^{2} }}{{\mu_{i} }}} \right)\;\;,\;i = E,\;R,\;P$$, where $$\gamma_{i1}$$ and $$\gamma_{i2}$$ represent the shape and scale parameters, respectively. The same simulation parameters such as $$\mu_{R} - \mu_{P}$$$$\tau_{R}$$,$$\tau_{P}$$,$$n$$ are the same as those in Simulation study I and II. The simulation results of the type I error rates are displayed in Tables 2 and 3, and the simulation results of empirical powers are presented in Table 4.

**Table 2:** Under Log-normal distribution, the type I error rates for testing non-inferiority with non-inferiority limit = 0.8 in $\tau_{R} = 1$, $\mu_{R} - \mu_{P} =$ 9, 15 and 20, respectively

Distribution	$\mu_{R} - \mu_{P}$	$\tau_{P}$	$n$	Method		
				GP	DM	EB
Log-Normal	9	0.5	60	0.0462	0.0002	0.0003
			90	0.0477	0.0002	0.0009
			120	0.0482	0.0003	0.0027
			480	0.0493	0.0027	0.0136
			900	0.0504	0.0036	0.0245
		1.0	60	0.0477	0.0006	0.0018
			90	0.0478	0.0008	0.0064
			120	0.0491	0.0010	0.0073
			480	0.0493	0.0012	0.0327
			900	0.0498	0.0015	0.0418
		2.0	60	0.0477	0.0001	0.0145
			90	0.0493	0.0002	0.0164
			120	0.0500	0.0005	0.0191
			480	0.0505	0.0008	0.0418
			900	0.0509	0.0011	0.0427
	15	0.5	60	0.0458	0.0020	0.0027
			90	0.0475	0.0023	0.0082
			120	0.0478	0.0029	0.0145
			480	0.0485	0.0047	0.0373
			900	0.0491	0.0054	0.0382
		1.0	60	0.0465	0.0013	0.0182
			90	0.0478	0.0016	0.0300
			120	0.0491	0.0019	0.0418
			480	0.0497	0.0020	0.0473
			900	0.0500	0.0023	0.0489
		2.0	60	0.0484	0.0002	0.0291
			90	0.0492	0.0003	0.0355
			120	0.0499	0.0005	0.0400
			480	0.0501	0.0012	0.0428
			900	0.0505	0.0015	0.0437
	20	0.5	60	0.0457	0.0038	0.0118
			90	0.0461	0.0046	0.0182
			120	0.0479	0.0055	0.0264
			480	0.0486	0.0059	0.0436
			900	0.0495	0.0065	0.0482
		1.0	60	0.0485	0.0011	0.0300
			90	0.0493	0.0023	0.0345
			120	0.0495	0.0026	0.0355
			480	0.0496	0.0030	0.0464
			900	0.0502	0.0040	0.0479
		2.0	60	0.0491	0.0003	0.0155
			90	0.0495	0.0007	0.0173
			120	0.0496	0.0010	0.0191
			480	0.0500	0.0013	0.0432
			900	0.0503	0.0015	0.0473

$n$ The total sample sizes, *GP* The GPV-based method, *DM* The Delta method, and *EB* the empirical bootstrap method

**Table 3:** Under Gamma distribution, the type I error rates for testing non-inferiority with non-inferiority limit = 0.8 in $\tau_{R} = 1$, $\mu_{R} - \mu_{P} =$ 9, 15 and 20, respectively

Distribution	$\mu_{R} - \mu_{P}$	$\tau_{P}$	$n$	Method		
				GP	DM	EB
Gamma	9	0.5	60	0.0489	0.0001	0.0001
			90	0.0497	0.0001	0.0009
			120	0.0500	0.0001	0.0036
			480	0.0506	0.0013	0.0236
			900	0.0509	0.0028	0.0373
		1.0	60	0.0493	0.0001	0.0009
			90	0.0501	0.0002	0.0027
			120	0.0506	0.0002	0.0045
			480	0.0512	0.0003	0.0209
			900	0.0516	0.0003	0.0273
		2.0	60	0.0486	0.0001	0.0109
			90	0.0495	0.0001	0.0136
			120	0.0499	0.0001	0.0191
			480	0.0505	0.0002	0.0424
			900	0.0509	0.0002	0.0451
	15	0.5	60	0.0483	0.0008	0.0027
			90	0.0499	0.0009	0.0036
			120	0.0496	0.0019	0.0082
			480	0.0510	0.0030	0.0300
			900	0.0513	0.0048	0.0364
		1.0	60	0.0485	0.0005	0.0145
			90	0.0499	0.0006	0.0173
			120	0.0502	0.0006	0.0273
			480	0.0509	0.0008	0.0409
			900	0.0514	0.0009	0.0418
		2.0	60	0.0495	0.0001	0.0282
			90	0.0496	0.0001	0.0318
			120	0.0506	0.0001	0.0427
			480	0.0511	0.0002	0.0433
			900	0.0516	0.0003	0.0472
	20	0.5	60	0.0482	0.0027	0.0073
			90	0.0500	0.0036	0.0164
			120	0.0505	0.0034	0.0191
			480	0.0508	0.0042	0.0305
			900	0.0509	0.0056	0.0436
		1.0	60	0.0487	0.0004	0.0200
			90	0.0500	0.0009	0.0227
			120	0.0501	0.0010	0.0400
			480	0.0508	0.0011	0.0418
			900	0.0515	0.0015	0.0482
		2.0	60	0.0484	0.0001	0.0318
			90	0.0486	0.0002	0.0409
			120	0.0500	0.0004	0.0415
			480	0.0509	0.0006	0.0435
			900	0.0515	0.0011	0.0484

$n$ The total sample sizes, *GP* The GPV-based method, *DM* The Delta method and *EB* The empirical bootstrap method

**Table 4:** Under non-normal distribution, the empirical powers of testing non-inferiority with non-inferiority limit = 0.8 in $\tau_{R} { = 1}$,$\tau_{P} = 2$

$n$	Distribution	Method	Empirical Power								
			$\mu_{R} - \mu_{P} = 9$			$\mu_{R} - \mu_{P} = 15$			$\mu_{R} - \mu_{P} = 20$		
			$\xi = 1.0$	$\xi = 1.2$	$\xi = 1.4$	$\xi = 1.0$	$\xi = 1.2$	$\xi = 1.4$	$\xi = 1.0$	$\xi = 1.2$	$\xi = 1.4$
60	Log-Normal	GP	0.3079	0.7160	0.8848	0.6654	0.9851	0.9989	0.8611	1.0000	1.0000
		DM	0.0152	0.0909	0.2677	0.0865	0.4726	0.8104	0.2192	0.7912	0.9597
		EB	0.1191	0.3382	0.5064	0.5418	0.9236	0.9418	0.8355	0.9945	0.9982
	Gamma	GP	0.3094	0.7365	0.9272	0.6693	0.9923	1.0000	0.8633	1.0000	1.0000
		DM	0.0028	0.0293	0.1402	0.0381	0.4006	0.8595	0.1407	0.8306	0.9954
		EB	0.0709	0.2855	0.5027	0.5600	0.9609	0.9891	0.8473	1.0000	1.0000
90	Log-Normal	GP	0.4379	0.8865	0.9702	0.8122	0.9998	0.9999	0.9557	1.0000	1.0000
		DM	0.0193	0.1580	0.4436	0.1358	0.6888	0.9431	0.3426	0.9290	0.9933
		EB	0.2055	0.5691	0.7400	0.7500	0.9873	0.9891	0.9527	1.0000	1.0000
	Gamma	GP	0.399	0.9124	0.9938	0.8187	1.0000	1.0000	0.9570	1.0000	1.0000
		DM	0.0049	0.0692	0.3275	0.0683	0.6989	0.9909	0.2578	0.9823	1.0000
		EB	0.1836	0.5627	0.7852	0.7491	1.0000	1.0000	0.9536	1.0000	1.0000
120	Log-Normal	GP	0.5436	0.9553	0.9905	0.9015	1.0000	1.0000	0.9871	1.0000	1.0000
		DM	0.0227	0.2394	0.5978	0.1973	0.8442	0.9850	0.4811	0.9832	0.9997
		EB	0.3073	0.7709	0.8609	0.8891	0.9982	1.0000	0.9836	1.0000	1.0000
	Gamma	GP	0.5501	0.9717	0.9912	0.9030	1.0000	1.0000	0.9880	1.0000	1.0000
		DM	0.0069	0.1382	0.5777	0.1279	0.9057	1.0000	0.4276	0.9990	1.0000
		EB	0.3082	0.8109	0.9173	0.8818	1.0000	1.0000	0.9818	1.0000	1.0000
480	Log-Normal	GP	0.9716	1.0000	1.0000	1.0000	1.0000	1.0000	1.0000	1.0000	1.0000
		DM	0.2765	0.9837	1.0000	0.9365	1.0000	1.0000	0.9993	1.0000	1.0000
		EB	0.9709	1.0000	1.0000	1.0000	1.0000	1.0000	1.0000	1.0000	1.0000
	Gamma	GP	1.0000	1.0000	1.0000	1.0000	1.0000	1.0000	1.0000	1.0000	1.0000
		DM	0.2349	0.9957	1.0000	0.9607	1.0000	1.0000	0.9999	1.0000	1.0000
		EB	0.9709	1.0000	1.0000	1.0000	1.0000	1.0000	1.0000	1.0000	1.0000
900	Log-Normal	GP	1.0000	1.0000	1.0000	1.0000	1.0000	1.0000	1.0000	1.0000	1.0000
		DM	0.7449	1.0000	1.0000	0.9999	1.0000	1.0000	1.0000	1.0000	1.0000
		EB	0.9991	1.0000	1.0000	1.0000	1.0000	1.0000	1.0000	1.0000	1.0000
	Gamma	GP	1.0000	1.0000	1.0000	1.0000	1.0000	1.0000	1.0000	1.0000	1.0000
		DM	0.7498	1.0000	1.0000	1.0000	1.0000	1.0000	1.0000	1.0000	1.0000
		EB	0.9982	1.0000	1.0000	1.0000	1.0000	1.0000	1.0000	1.0000	1.0000

$n$ The total sample sizes, *GP* The GPV-based method, *DM* The Delta method and *EB* The empirical bootstrap method

From Tables 2 and 3, when data follow log-normal or gamma distribution, the performance of GPV-based method can more appropriately maintain the type I error rate near the nominal level of 0.05 than the Delta method and the empirical bootstrap method do. In addition, the type I error rate of the Delta method is quiet conservative as well. Furthermore, under $\mu_{R} - \mu_{P} = 20$, $\tau_{R} = 1$, $\tau_{P} = 2$ and the total sample size is greater than 900, the type I error rate derived from the empirical bootstrap method approaches the claimed significance level of the non-inferiority test. Moreover, in Table 4, regardless of the sample size and distributions, the empirical power performance of GPV-based method is more powerful than that of the Delta method and the empirical bootstrap method, especially under the $\mu_{R} - \mu_{P} = 9$, $\tau_{R} = 1$, $\tau_{P} = 2$ and the total sample size is less than 120.

### Numerical example: evaluation of the mutagenicity

We adopt the mutagenicity data set in Hauschke et al. [21], which are published by Adler and Kliesch [22] from a micronucleus assay on hydroquinone implementing a positive control of 25 mg/kg cyclophosphamide. The results for male mice at 24 h sampling time are given in Table 5.

**Table 5:** Summary statistics for the number of micronuclei per animal and 2000 scored cells for the vehicle control, four doses of hydroquinone and the positive control of 25 mg/kg cyclophosphamide

Treatment group	Mean	Standard deviation	Sample size
Vehicle control	2.57	1.27	7
30 mg/kg	3.80	1.10	5
50 mg/kg	6.20	1.48	5
75 mg/kg	14.0	3.94	5
100 mg/kg	20.0	4.06	5
Positive control	25.0	8.91	4

Through comparing the difference between a dose group and a vehicle control with the difference between the positive control and the vehicle control, the non-inferiority test can also be adopted to verify the safety in toxicological experiments. Therefore, the above mutagenicity data can be evaluated by such non-inferiority test. Hothorn and Hauschke [23] used the concept of the acceptable maximal safe dose by identifying the highest dose that is non-inferior to the vehicle control, and as a result all other levels of dose below the highest one are also non-inferior. Under the assumption of normality and homogeneous variance, Hauschke et al. [21] built confidence intervals for the ratio of the difference between the dose groups and the vehicle control to the difference between a positive control and the vehicle control, in which the safety threshold is set to be 0.5. Hence, the hypothesis of the corresponding non-inferiority test can be characterized as follows.

14 $$H_{0} :\frac{{\theta_{E} - \theta_{P} }}{{\theta_{R} - \theta_{P} }} \ge 0.5{\kern 1pt} {\kern 1pt} {\text{versus}}{\kern 1pt} {\kern 1pt} H_{0} :\frac{{\theta_{E} - \theta_{P} }}{{\theta_{R} - \theta_{P} }} < 0.5$$

where the dose group is taken as the experimental group, the vehicle control taken as the placebo group and the positive control taken as the reference group. The upper 95% confidence limits for $\frac{{\theta_{E} - \theta_{P} }}{{\theta_{R} - \theta_{P} }}$ calculated from GPV-based method, the Delta method, and the empirical bootstrap method are presented Table 6.

**Table 6:** Upper 95% confidence limits for $\frac{{\theta_{E} - \theta_{P} }}{{\theta_{R} - \theta_{P} }}$, based on the positive control of 25 mg/kg cyclophosphamide

Treatment group	*P*-value	Upper confidence limit		
		GPV-based method	Delta method	Empirical bootstrap method
30 mg/kg	0.0018	0.28	0.13	0.08
50 mg/kg	0.0044	0.41	0.29	0.23
75 mg/kg	0.2474	0.97	0.74	0.74
100 mg/kg	0.8566	1.39	1.06	1.13

From Table 6, one can see that safety is attainable for the two lower doses, therefore the maximal safe dose is 50 mg/kg. The two higher levels of dose, 75 and 100 mg/kg, reveal an unacceptable increase. Cases where the variance heterogeneity is taken into account in the GPV-based method, the Delta method, and the empirical bootstrap method, the results do not change.

## Conclusions and discussions

We propose the GPV-based method to conduct the non-inferiority test by the difference of means with unknown coefficient of variations between the experimental and the placebo groups relative to that between the reference and the placebo groups under the normality assumption. The main contribution of this research is that we revise the measurement of non-inferiority by considering the coefficient of variation (CV) of each kind of treatment from the average effect of trials. This is slightly different from the traditional non-inferiority test that is difference of means between the experimental and the placebo groups relative to that between the reference and the placebo groups. Besides, through the heuristic statistical testing procedure for non-inferiority test, we incorporate unknown heterogeneous variance among the three arms. Hence, CVs are included in the non-inferiority hypothesis testing explicitly to help prevent possible estimating distortion if heteroskedasticity is allowed.

Empirical results from simulation studies show that the GPV-based method can not only adequately control the type I error rates at the nominal level but also provide power higher than those from the Delta method and the empirical bootstrap method. The performances of empirical type I error rates and empirical power of GPV-based method are better than those from the Delta method and the empirical bootstrap method. Therefore, the GPV-based method is suitable to conduct the non-inferiority test for the means with unknown coefficient of variation in a three-arm trial. The R program for the proposed GPV-based method is available as Supplementary materials 1 and 2.

To further explore the properties of these comparable methods, estimations are conducted for non-inferiority limit under parameter settings as in simulation studies. The non-inferiority limit is chosen as 0.8. For each specified parameter combination, the data are generated 10,000 times independently. The bias, mean square error (MSE) and coverage probability (CP) simulation results of the three methods are shown in Table 7.

**Table 7:** Under $\tau_{R} - \tau_{P}$ = 9, 15, and 20, estimate the Bias, MSE and CP of non-inferiority limit by the GPV-based, the Delta, and the empirical bootstrap methods

$\mu_{R} - \mu_{P}$	$\tau_{P}$	$n$	Point Estimation Property						Interval Estimation Property		
			Bias			MSE			CP		
			GP	DM	EB	GP	DM	EB	GP	DM	EB
9	0.5	60	0.0340	0.0865	0.2170	0.1114	5.7267	53.2988	0.9503	0.9999	0.9999
		90	0.0246	0.0407	0.1125	0.0815	1.1064	8.8637	0.9497	0.9999	0.9997
		120	0.0221	0.0295	0.0847	0.0595	0.0712	3.2183	0.9494	0.9998	0.9991
		480	0.0080	0.0080	0.0130	0.0123	0.0123	0.0276	0.9489	0.9984	0.9918
		900	0.0015	0.0015	0.0057	0.0063	0.0063	0.0133	0.9482	0.9962	0.9736
	1.0	60	0.0186	0.0240	0.0133	0.0590	0.5243	15.3224	0.9514	0.9999	0.9985
		90	0.0139	0.0152	0.0400	0.0393	0.0467	1.7485	0.9507	0.9998	0.9982
		120	0.0135	0.0141	0.0266	0.0283	0.0295	0.2401	0.9502	0.9998	0.9955
		480	0.0027	0.0028	0.0022	0.0061	0.0061	0.0129	0.9499	0.9997	0.9682
		900	0.0011	0.0011	0.0013	0.0033	0.0033	0.0067	0.9494	0.9996	0.9564
	2.0	60	0.0098	0.0097	0.0260	0.0300	0.0446	2.4935	0.9512	0.9999	0.9909
		90	0.0067	0.0058	0.0051	0.0196	0.0206	0.1298	0.9501	0.9999	0.9873
		120	0.0058	0.0053	0.0024	0.0143	0.0145	0.0421	0.9496	0.9998	0.9736
		480	0.0012	0.0011	0.0008	0.0033	0.0033	0.0068	0.9494	0.9998	0.9618
		900	0.0011	0.0011	0.0004	0.0018	0.0018	0.0035	0.9489	0.9997	0.9564
15	0.5	60	0.0226	0.0231	0.0465	0.0400	0.0431	0.2712	0.9525	0.9992	0.9982
		90	0.0151	0.0157	0.0306	0.0255	0.0257	0.0691	0.9519	0.9991	0.9945
		120	0.0113	0.0117	0.0177	0.0189	0.0189	0.0897	0.9518	0.9981	0.9909
		480	0.0017	0.0017	0.0034	0.0044	0.0044	0.0087	0.9501	0.9964	0.9727
		900	0.0005	0.0005	0.0025	0.0023	0.0023	0.0045	0.9498	0.9957	0.9636
	1.0	60	0.0124	0.0113	0.0201	0.0192	0.0195	0.0799	0.9507	0.9996	0.9845
		90	0.0078	0.0073	0.0130	0.0126	0.0126	0.0277	0.9499	0.9996	0.9809
		120	0.0045	0.0042	0.0087	0.0093	0.0094	0.0195	0.9495	0.9993	0.9782
		480	0.0011	0.0011	0.0012	0.0022	0.0022	0.0044	0.9492	0.9993	0.9577
		900	0.0008	0.0008	0.0013	0.0012	0.0012	0.0023	0.9486	0.9985	0.9555
	2.0	60	0.0053	0.0041	0.0094	0.0095	0.0095	0.0224	0.9500	0.9999	0.9673
		90	0.0031	0.0028	0.0026	0.0064	0.0064	0.0133	0.9497	0.9998	0.9636
		120	0.0014	0.0009	0.0013	0.0048	0.0048	0.0102	0.9496	0.9998	0.9627
		480	0.0003	0.0003	0.0009	0.0012	0.0012	0.0023	0.9493	0.9997	0.9545
		900	0.0002	0.0002	0.0007	0.0006	0.0006	0.0013	0.9488	0.9996	0.9527
20	0.5	60	0.0145	0.0135	0.0251	0.0221	0.0223	0.0514	0.9505	0.9979	0.9927
		90	0.0074	0.0070	0.0144	0.0139	0.0139	0.0291	0.9497	0.9975	0.9873
		120	0.0044	0.0041	0.0092	0.0099	0.0099	0.0212	0.9489	0.9968	0.9836
		480	0.0010	0.0010	0.0034	0.0024	0.0024	0.0050	0.9486	0.9953	0.9645
		900	0.0006	0.0006	0.0013	0.0012	0.0012	0.0026	0.9484	0.9942	0.9536
	1.0	60	0.0072	0.0063	0.0106	0.0101	0.0101	0.0221	0.9513	0.9994	0.9736
		90	0.0042	0.0038	0.0068	0.0066	0.0066	0.0141	0.9507	0.9990	0.9709
		120	0.0036	0.0034	0.0044	0.0050	0.0050	0.0104	0.9496	0.9990	0.9697
		480	0.0001	0.0001	0.0006	0.0012	0.0012	0.0024	0.9490	0.9988	0.9545
		900	0.0002	0.0002	0.0002	0.0007	0.0007	0.0013	0.9487	0.9985	0.9509
	2.0	60	0.0039	0.0031	0.0019	0.0053	0.0053	0.0112	0.9514	0.9999	0.9582
		90	0.0030	0.0026	0.0014	0.0035	0.0035	0.0072	0.9510	0.9999	0.9564
		120	0.0019	0.0017	0.0012	0.0026	0.0026	0.0054	0.9498	0.9998	0.9555
		480	0.0002	0.0001	0.0005	0.0006	0.0006	0.0013	0.9491	0.9997	0.9536
		900	0.0002	0.0002	0.0003	0.0003	0.0003	0.0007	0.9485	0.9995	0.9523

$n$ The total sample sizes, *GP* The GPV-based method, *DM* The Delta method and *EB* The empirical bootstrap method, *MSE* Mean square error, *CP* coverage probability

From Table 7, the biases from the GPV method are not much different to those from Delta method, but most of which are smaller than the empirical bootstrap method. Furthermore, when the mean difference of the reference and placebo groups is equal to 9 and sample size is less than 120, one can see that the GPQ from GPV-based method has smaller MSE than estimators from the Delta method and the empirical bootstrap method do. On the other hand, the GPV-based method generally provides sufficient coverage probabilities around the confidence level of 0.95. The GPV-based method approach results in fairly better coverage probability than the other two methods do, regardless of the sample size. Moreover, when the mean difference of reference and placebo groups is large than 20, under the ratio of variance of the reference group to the experimental group is 1 and the ratio of variance of the placebo group to the experimental group is 2, the performances of coverage probabilities of the empirical bootstrap method are as good as that of the GPV-based method. Additionally, the coverage probabilities presented by the Delta method are quite conservative as well.

Under the normality assumption, the required percentiles of GPQ for $\frac{{\theta_{E} - \theta_{P} }}{{\theta_{R} - \theta_{P} }}$ (our measurement of non-inferiority) cannot be obtained in closed form but may be estimated using Monte-Carlo algorithm. In addition, if the data belongs to non-normal data, we recommend that the power transformation of Box and Cox [24] be performed.

In Wu and Hsieh [5], when conducting non-inferiority test in a three-arm trial, they estimate the sample mean by Searls’ estimator (mean with CV) rather than the traditional one (pure sample mean), showing that testing results are better, in terms of empirical sizes and empirical powers. While in our research, different from the traditional three-arm trial, we conduct the non-inferiority test for the means with unknown CVs, and we show that the explicit inclusion of CVs in the measurement of non-inferiority can still control the type I error at the nominal level. In sum, when conducting non-inferiority test, CVs are highly recommended to be included, whether through the estimation of average effects of trials or through the specification of non-inferiority.

### Supplementary Information

**Additional file 1. **

**Additional file 2. **

## Data Availability

The numerical example used and analyzed during this study may be obtained from the corresponding author on reasonable request.

## Abbreviations

GP: The GPV-based method
DM: The Delta method
EB: The empirical bootstrap method
*n*: The total sample sizes
